# A cross-sectional hospital-based study of correlates of disability in patients with chronic low back pain in KwaZulu-Natal, South Africa

**DOI:** 10.1186/s12891-022-05397-4

**Published:** 2022-05-11

**Authors:** Morris Kahere, Themba Ginindza

**Affiliations:** Discipline of Public Health Medicine, School of Nursing and Public Health, University of KwaZulu-Natal, 2nd Floor George Campbell Building, Mazisi Kunene Road, Durban, 4041 South Africa

**Keywords:** Chronic low back pain, Disability, Fear avoidance beliefs, Illness behavior, Risk factors

## Abstract

**Background:**

Chronic low back pain (CLBP) is the leading cause of disability globally and is a major concern in public health. However, there is limited evidence on the prevalence and correlates of disability among adults in Sub-Saharan Africa. Thus, this study aimed at determining factors influencing disability among adult patients with CLBP in KwaZulu-Natal.

**Methods:**

This analytical cross-sectional hospital-based study was conducted among adult CLBP patients in KwaZulu-Natal, South Africa. Data on disability, fear avoidance beliefs and illness behavior were gathered from 554 adult participants using self-administered questionnaires. Multiple linear regression analysis was conducted to determine factors associated with disability. Statistical significance was set at *p* < 0.05.

**Results:**

Based on the multivariable linear regression, being a female (β = 0.343, *p* < 0.001) and fear avoidance beliefs about work (β = 0.221, p = 0.044) were significantly associated with greater disability, while, smoking 1 to 10 cigarettes per day (β = -0.106, *p* = 0.011) and higher illness behaviour scores (β = -0.165, *p* = 0.024) were significantly associated with less disability The model accounted for 20% of the total variance in Oswestry disability scores.

**Conclusion:**

This study has concluded that disability in CLBP is predicted by multiple of factors, with psychosocial factors (fear avoidance beliefs and heavy cigarette smoking) playing a significant role. Manual work was also identified as a significant predictor of CLBP disability. Therefore, guidelines should emphasize on early identification of these yellow flags in primary care.

**Supplementary Information:**

The online version contains supplementary material available at 10.1186/s12891-022-05397-4.

## Background

Chronic low back pain (CLBP) is a complex and multifactorial musculoskeletal disorder (MSKD) that has become the leading cause of disability worldwide, associated with high societal and health economic costs [1, 2]. While the experience of low back pain (LBP) is common, and many cases resolving within a few days or weeks with little or no attention, some presentation will have episodes of chronic recurrent pain and disability (limitations in activities of daily living) [3]. The majority of disability and financial burden associated with LBP is attributed to those who develop CLBP (LBP persisting for ≥ 12 weeks) [4]. According to the Global Burden of Disease 2017, the years lived with disability (YLD) due to LBP increased by 52.7%, from 42.5 million in 1990 to 64.9 million in 2017 [5]. In 2019, the global LBP prevalent cases were 568.4 million, with an age-standardized point-prevalence of 6972.5 per 100,000 population and 223.5 million incidence cases with an age-standardized annual incidence of 2748.9, globally [6]. The global prevalence of CLBP is estimated at 19.6%, globally [7] and is expected to increase in low-and-middle-income-countries (LMICs), where the healthcare systems are already overstretched by the burden of life-threatening communicable diseases [8, 9].

Musculoskeletal disorders contribute about 3.4% and 1.7% of the total global burden of disease in high-income-countries and low-and-middle-income-countries, respectively [10]. For MSKD in LMIC, the survival rates are significantly lower compared to those reported in HICs such as USA, Western Europe, and Canada where 5-year and 10-year survival rates surpass 90% [11]. In South Africa (SA), the 5-year survival rate due to MSKD was shown to be between 57 and 72% (1986—2003) in a case series of 226 patients [11]. Evidence has shown that MSKD are the major causes of global disability and chronic-ill-health and further indicates a wide gap between HICs and LMICs, with osteoarthritis and CLBP remaining the largest contributors [12, 13]. The prevalence of CLBP in SA has been reported in Cape Town and KwaZulu-Natal and was estimated to be 26.3% and 18.1%, respectively [14, 15]. A study on MSKD among nurses in two South African hospitals reported that there is a high burden of MSKD in South Africa, with CLBP ranking the top in terms of prevalence and disability [16].

With the advent of the 4^th^ industrial revolution, recent technological advances in medical diagnostics have contributed significantly to elaborate the pathogenic mechanisms of chronic pain progression. This has led to early detection of mild disease and the inventions of new therapies which have resulted in improved outcomes in HICs [13]. However, despite these advances in medical technology and the observed improved outcomes, MSKD still rank the highest in prevalence and cause of chronic-ill-health and disability globally [10]. The burden of CLBP has been projected to continue increase especially in LMICs, where the healthcare and social systems are not equipped enough to deal with the increasing burden due to other competing healthcare priorities of communicable diseases [8]. International guidelines such as the sustainable development goals (SDG) aim to improve health outcomes in SSA, therefore evidence on disease burden is urgently needed to influence policy implementation and enhance knowledge-based decision making. Although CLBP is regarded as a trivial, self-limiting, rarely fatal condition, it’s socioeconomic burden will remain significantly high if no action is taken.

South Africa’s National Development Plan and health policy seek to reduce the burden of non-communicable diseases and improve health outcomes, yet correlated of CLBP disability, the leading driver of disability are still unknown in this context [17]. However, notwithstanding the projected increase in CLBP burden, there is severe lack of evidence on factors associated with CLBP disability in SA. In Sub-Saharan Africa, the few studies found after an extensive search of literature that investigated factors associated with CLBP disability were conducted in Cameroon and Nigeria by Doualla et al. and Igwesi-Chidobe et al., respectively [18, 19]. Doualla et al. reported that pain intensity, longer days of work absence, and bladder/bowel dysfunction symptoms were associated with greater disability, while alcohol consumption and higher psychological well-being scores were associated with less disability [19]. Igwesi-Chidobe et al. reported that abnormal illness perception, pain intensity, catastrophizing, FAB and anxiety were significantly associated with self-reported disability while abnormal illness perception, lack of social support, fear avoidance beliefs and the female sex were associated with performance based disability [18]. According to Igwesi-Chidobe et al. occupational biomechanical factors did not predict disability in CLBP patients [18].

Given the multifactorial nature of CLBP and its predominant psychosocial predisposition, the traditional biomedical model of management has shown limited effectiveness, thus evidence suggests that interventions should focus on a patient centered holistic biopsychosocial approach [20]. Therefore, identification of these psychosocial predictors is an important initial step to effective management. Studies from HICs have recommended routine screening of these yellow flags (psychosocial risk factors) in primary health care (PHC). Against this background, a risk stratification tool called STarT Back was developed in the United Kingdom (UK) for use as part of the initial assessment in PHC [21]. This tool was then adapted, translated and validated for us in isiZulu, a common South African local language [22]. Even though, there is evidence on CLBP disability from HICs, Central Africa (Cameroon) and West Africa (Nigeria), nothing is known from the South of Africa. Extrapolating results of studies from these contexts for use in South Africa is difficult due to differences in health care and social systems, insurance coverage (health financing) and legislations. Therefore, context specific data is needed to develop culturally validated guidelines. It is against this backdrop that we conducted this case study in KwaZulu-Natal (KZN) to understand the factors associated with disability among adult LBP patients.

## Materials and methods

### Study design

This is part of a larger study that sought to determine the burden of CLBP among adults in KwaZulu-Natal. This was a cross-sectional, hospital-based study that was conducted by means of self-report questionnaires at primary public hospitals in the eThekwini district municipality of KwaZulu-Natal province in South Africa. This study was conducted in accordance with the Declaration of Helsinki and was reported in compliance with the STrengthening the Reporting of OBservational studies in Epidemiology (STROBE) checklist and explanation [23, 24].

### Study area and setting

This study was conducted at the following primary public hospitals, namely: Prince Mshiyeni Memorial Hospital, Mahatma Gandhi Memorial, Clairwood, Hillcrest, and Addington in December 2019. The South African primary public healthcare is organized into primary health care centers (clinics, mobile clinics) and community health centers which provides health services to the majority of the population through a hierarchical referral health system where district and provincial hospitals provide tertiary care. eThekwini municipality, that includes the city of Durban, is the only metropolitan and most populous of the eleven municipalities in KwaZulu-Natal. It has a population of 3 158 000 inhabitants and has 18 provincial hospitals.

### Participantsand sampling

The study investigated 554 adult males and females (aged ≥ 18 years) with self-reported current and or prior history of low back pain, attending outpatient-care at the selected study sites for health-related services. Participants were recruited using a systematic random sampling technique, (after the first participant was selected using the lottery method) where every third patient presenting to the clinic was invited to participate until the required sample size was achieved. Eligible participants that were willing to participate and sign informed consent were enrolled into the study. The purpose and procedures of the research was then explained in detail to the enrolled participants. Participation in this study was voluntary, and all participants were pre-framed that they can discontinue at any time should they wish to do so. Each participant was given a set of self-report close ended questionnaires (including the Oswestry low back pain disability questionnaire) to fill out. Data was collected once off, without follow-up or repeated measures. All completed questionnaires were handed back to the research assistants for error checking and data capturing. Low back pain was defined as sensations of pain, muscle tension, or stiffness, localized below the costal margin and above the inferior gluteal folds with or without leg pain, and as chronic if the symptoms persists for 12 weeks and above.

### Study eligibility criteria

The study included adult (aged ≥ 18 years) LBP patients who presented at any of the participating hospitals during the study period for health-related services and were willing to sign informed consent. We excluded children aged below 18 years, and those that were not able to give consent (such as mentally disabled), and those with severe chronic diseases other than LBP.

### Sample size

As part of a larger study that was conducted to determine the burden of chronic low back pain among adults in KwaZulu-Natal, this study utilized the same sample size estimation, elaborated next. To estimate the prevalence of the outcomes of interest (chronic low back pain), assuming 95% confidence and an acceptable margin of error of 5%, and maximum variability i.e., 50% (given unknown prevalence) a sample size of 384 subjects was required. The sample was further increased by a margin of 10% to account for potential non-response and multiplied by a design effect (D) of 1.3. The final sample size of the entire study was 650 participants. We considered the methodology of the study described in the published protocol [25]. However, based on the inclusion criteria of this portion of the study, only 554 participants satisfied the criteria and were selected for further analysis.

### Study instruments

Data on demographic characteristics of participants were gathered using a self-reported questionnaire and included age, sex, marital status, body mass index, educational level, income category, exercise frequency, smoking attitude, alcohol consumption, employment type and sitting posture. Other clinical characteristics included LBP severity (mild, moderate or severe), presence of sciatica/leg pain (yes or no).

Low back pain disability was measured using the Oswestry low back pain disability questionnaire [26]. The OSD is validated, reliable and has high internal consistency (Cronbach α) of 0.87 [26, 27]. It is comprised of 10 sections each with a total possible score of 5 and the overall score is expressed as a percentage. A score of 0 – 20% is considered minimal disability where patients can cope with most daily living activities without treatment apart from ergonomic advice. A score of 21%—40% is considered moderate disability and indicates that, the patient experiences pain and disability with most activities of daily living. Participants scoring 41%—60% were considered severely disabled. These participants have pain as their main problem and require special detailed investigations. Participants scoring 61%—80% were considered crippled, indicating significant impact to activities of daily living and requires detailed investigation and positive intervention. Participants scoring 81%—100% on the OSD are either bed-bound or exaggerating their symptoms.

Fear-avoidance belief was measured using the Fear Avoidance Belief Questionnaire (FABQ) [28]. The FABQ is a validated, reliable self-report 16 item, 7-point Likert-scale questionnaire with item scores ranging between 0 (strongly disagree) and 6 (strongly agree) where scores are directly proportional to the level of fear avoidance belief. The FABQ is comprised of two subscales: beliefs about work and beliefs about physical activity [29].

Illness behavior was measured using the Pilowsky and Spence 62-item Illness Behavior Questionnaire (IBQ) (27). The IBQ consist of at binary response items (Yes/No), where “Yes” is given a score of 1 and “No” a score of 0. The IBQ’s highest total possible score is 62 and the lowest possible score is zero (0). Higher scores indicate abnormal illness behavior. A detailed presentation of IBQ’s validity and reliability is provided in the Manual for the Illness Behavior Questionnaire (29).

Occasional alcohol consumption was defined as at least 5 standard drinks for not more than twice per week and frequent consumption was defined as at least 5 standard drinks for at least three time per week [30]. Frequent exercise was defined as moderate to vigorous-intensity exercise ≥ 30 min for at least 5 times per week [31]. A self-report of 1 – 3 numerical pain rating scale was considered mild, 4 – 6 was considered moderate while 7 – 10 was severe [32]. Semi-sedentary type of work involved both sitting down and standing for prolonged periods of time, while sedentary referred to those involved in occupations that demands them to sit for 6 – 8 h per day. Manual labour was defined as lifting of heavy objects or any manual type of work. In terms of the body mass index, < 18.5 was considered underweight, 18.5 – 24.9 was considered normal, 25.0 – 29.9 was overweight and ≥ 30.0 was considered obese [33]. According to Maphupha (2018), the income categories (in South African Rand, ZAR) were defined as poor (0 – 54 344), low emerging middle (54 345 – 151 727), emerging middle (151 728 – 363 930), realized middle (363 931 – 631 120) and upper middle 631 212 – 863 906) [34].

### Statistical analysis

The IBM Statistical Package for Social Sciences (SPSS) software version 27.0 for Windows (IBM Corp., Armonk, NY, USA) was used for data cleaning and descriptive statistics. STATA® 17.0 SE (Stata corp. College station, Texas, USA) was used for inferential statistical analysis. Participants sociodemographic characteristics were presented as frequencies (n) and proportions (%) for categorical variables. We presented continuous variables as mean ± standard deviation. The continuous score of the OSD was set as the dependent variable. Pearson’s correlation was used to assess the association between OSD and continuous independent variables (illness behavior scores and fear avoidance belief scores). The independent samples t-test was used for dichotomous independent variables while the analysis of variance (ANOVA) was used for independent variables with more than two categories. Variables that were significant at a significant cut-off (*p* < 0.2) in bivariate analysis, were selected for inclusion in the linear regression model that was initially performed univariately. To determine factors that were independently associated with disability, a multivariate linear regression analysis was performed using a stepwise backward selection method and included only the variables that were significant at a significant cut-off of *p* < 0.2 in the univariate analysis. Prior to performing the linear regression analysis standard assumptions of parametric tests (i.e. dependent and independent variables measured at a continuous scale, linearity, normality of residuals, homoscedasticity) were tested and satisfied. Evidence of multicollinearity in the independent variables was checked via a correlation matrix and then collinearity diagnostics was done to assess the tolerance and variance inflation factors (VIF). All the VIF were less than 4, indicating absence of any multicollinearity. The level of significance was set at *p* < 0.05.

## Results

A total of 554 LBP participants comprising 326 (58.8%) females and 228 (41.2%) males participated in the study (Table 1). The male to female ration was almost 2:3 (41.2%: 58.8%). The mean (± standard deviation) age for the participants was 45.8 (± 10.7 years), and 15.3% of the participants were aged above 58 years. Almost half of the participants were married (48.0%). The prevalence of CLBP was 22.2%. Chronic low back pain was observed in 38.1% and 58.7% of those who self-reported moderate and severe symptoms, respectively. The prevalence of sciatica was 19.1%. Table 2 illustrates the stratified prevalence of non-chronic and chronic low back pain. The mean (± standard deviation) for OSD was 41.2 ± 10.9 (the 25^th^ – 75^th^ percentile = 34 – 48), which is moderate to severe disability. We have also conducted an age stratified descriptive analysis (supplementary file), which showed that the prevalence of smoking was highest at the middle-aged adults (38 – 47).

**Table 1:** Descriptive statistics

**Variable**	**Overall (*N* = 554)**		**Non-chronic Low Back Pain (*n* = 431)**		**Chronic Low Back Pain (*n* = 123)**	
	**n**	**%**	**n**	**%**	**n**	**%**
**Age (***Mean* ± *SD***)**	45.8 ± 10.7					
18 – 27	19	3.4	13	3.0	6	4.9
28 – 37	111	20.0	90	20.9	21	17.1
38 – 47	176	31.8	141	32.7	35	28.5
48 – 57	163	29.4	129	29.9	34	27.6
58 +	85	15.3	58	13.5	27	22.0
**Gender**						
Male	228	41.2	183	42.5	45	36.6
Female	326	58.8	248	57.5	78	63.4
**Marital status**						
Single	80	14.4	60	13.9	20	16.3
Married	266	48.0	201	46.6	65	52.8
Separated	157	28.3	129	29.9	28	22.8
Widowed	51	9.2	41	9.5	10	8.1
**Body Mass Index**						
Underweight	51	9.2	44	10.2	7	5.7
Normal	166	30.0	154	35.7	12	9.8
Overweight	202	36.5	140	32.5	62	50.4
Obese	135	24.4	93	21.6	42	34.1
**Education level**						
No formal education	106	19.1	74	17.2	32	26.0
Primary	194	35.0	164	38.1	30	24.4
Secondary	144	26.0	121	28.1	23	18.7
Tertiary	110	19.9	72	16.7	38	30.9
**Income category**						
Poor	104	18.8	68	15.8	36	29.3
Low emerging middle	273	49.3	224	52.0	49	39.9
Emerging middle	96	17.3	78	18.1	18	6.5
Realised middle	49	8.8	39	9.0	10	8.1
Upper middle	32	5.8	22	5.1	10	8.1
**Exercise**						
No	338	61.0	245	56.8	93	75.6
Yes	216	39.0	186	43.2	30	24.4
**Cigarette smoking**						
No	324	58.5	294	68.2	30	24.4
Yes, 1 – 10 per day	126	22.7	94	21.8	32	26.0
Yes. More than 11	104	18.8	43	10.0	61	49.6
**Alcohol consumption**						
No	264	47.7	234	54.3	30	24.4
Occasional	148	26.7	118	27.4	30	24
Frequent	142	25.6	79	18.3	63	51.2
**Type of work**						
Semisedentary	311	56.1	301	69.8	10	8.1
Sedentary	80	14.4	37	8.6	43	35.0
Manual work	163	29.4	93	21.6	70	56.9
**Sitting posture**						
Straight back	58	10.5	39	9.1	19	15.4
Stooped	106	19.1	66	15.3	40	32.5
Forward inclination	189	34.1	138	32.0	51	41.5
Backward inclination	201	36.3	188	43.6	13	10.6
**LBP severity**						
Mild	303	54.7	297	68.9	6	4.9
Moderate	147	26.5	91	21.1	56	45.5
Severe	104	18.8	43	10.0	61	49.6
**Sciatica**						
Yes	106	19.1	84	19.5	22	17.9
**No**	448	80.9	347	80.5	101	82.1
**Illness behaviour (***Mean* ± *SD***)**	36.6 ± 11.4		32.36 ± 9.2		51.6 ± 2.7	
**Fear avoidance belief**						
Work subscale (*Mean* ± *SD*)	26.3 ± 7.2		23.15 ± 4.5		37.3 ± 1.9	
Physical activity subscale (*Mean* ± *SD*)	11.1 ± 4.3		9.13 ± 2.2		18.1 ± 1.9	
**Disability (OSD score mean ± SD)**	41.2 ± 10.9		40.12 ± 10.5		45.1 ± 11.2	

*SD* Standard Deviation

**Table 2:** Stratified analysis of the prevalence of chronic and non-chronic low back pain

Age category	Non-Chronic Low Back Pain (*n* = 431)				Chronic Low Back Pain (*n* = 123)			
	Mean OSD ± SD	(n)	(%)	95% CI	Mean OSD ± SD	(n)	(%)	95% CI
**18 – 27**	44.8 ± 9.2	13	3.0	1.6 – 5.1	49.7 ± 13.8	6	4.9	1.8 – 10.3
**28 – 37**	39.4 ± 10.0	90	20.9	17.1 – 25.0	43.9 ± 10.3	21	17.1	10.9 – 24.9
**38 – 47**	39.1 ± 10.8	141	32.7	28.3 – 37.4	44.7 ± 10.1	35	28.5	20.7 – 37.3
**48 – 57**	41.8 ± 10.2	129	29.9	25.6 – 34.5	45.4 ± 12.1	34	27.6	19.7 – 36.5
**58 + **	38.8 ± 11.3	58	13.5	10.4 – 17.1	45.1 ± 12.6	27	22.0	14.9 – 30.3

Results of the bivariate analysis (Table 3) showed that sex, cigarette smoking, type of work, LBP duration and severity were significantly associated with disability. We further employed the multiple comparison Bonferroni post-hoc test for significance. Our findings indicated that the mean OSD scores for smoking more than 11 cigarettes per day (43.9 ± 11.2) was significantly higher than smoking 10 cigarettes or less per day (39.5 ± 11.4), Bonferroni-*p* = 0.007. Therefore, heavy smokers were associated with severe disability, requiring specific intervention while light smokers were associated with moderate disability, meaning that they experienced pain and disability in most activities of daily living. Similarly, the OSD score mean was significantly higher in manual work (42.8 ± 10.9) when compared to semi-sedentary (40.0 ± 10.4), Bonferroni-*p* = 0.021. Therefore, manual work was significantly associated with severe disability, with pain as the main problem and requiring special detailed investigation. The Pearson’s correlation (Table 4) indicated that illness behavior (*r* = 0.166, *p* = 0.006) and fear avoidance beliefs about work (*r* = 0.20, *p* < 0.001) and physical activity (*r* = 0.19, *p* < 0.001) had weak positive correlations with disability.

**Table 3:** OSD score variations by sociodemographic and clinical characteristics

Variable	OSD score mean ± SD	t or F statistics	*p*-value
**Age**			
18 – 27	46.3 ± 10.7	2.28	0.059
28 – 37	40.3 ± 10.1		
38 – 47	40.2 ± 10.9		
48 – 57	42.6 ± 10.7		
58 +	40.9 ± 11.9		
**Gender**			
Male	45.7 ± 11.0	8.65	0.000**
Female	38.1 ± 9.7		
**Marital status**			
Single	43.0 ± 11.5	1.27	0.284
Married	41.4 ± 10.8		
Separated	40.6 ± 10.7		
Widowed	39.6 ± 11.1		
**Body Mass Index**			
Underweight	41.0 ± 9.9	1.32	0.266
Normal	40.0 ± 10.6		
Overweight	42.2 ± 11.3		
Obese	41.4 ± 10.9		
**Education level**			
No formal education	42.4 ± 12.0	0.69	0.559
Primary	41.3 ± 10.6		
Secondary	40.5 ± 11.2		
Tertiary	40.9 ± 9.9		
**Income category**			
Poor	40.7 ± 10.6	0.645	0.628
Low emerging middle	41.0 ± 11.0		
Emerging middle	41.3 ± 11.2		
Realised middle	41.6 ± 10.9		
Upper middle	44.1 ± 10.2		
**Exercise**			
No	41.9 ± 11.2	1.95	0.052
Yes	40.1 ± 10.3		
**Cigarette smoking**			
No	41.1 ± 10.4	4.83	0.008*
Yes, 1 – 10 per day	39.5 ± 11.4		
Yes. More than 11	43.9 ± 11.2		
**Alcohol consumption**			
No	41.1 ± 10.4	0.79	0.454
Occasional	40.6 ± 11.5		
Frequent	42.2 ± 11.2		
**Type of work**			
Semisedentary	40.0 ± 10.4	4.98	0.007*
Sedentary	43.0 ± 12.0		
Manual work	42.8 ± 10.9		
**Sitting posture**			
Straight back	43.7 ± 10.3	2.45	0.063
Stooped	42.6 ± 11.4		
Forward inclination	40.0 ± 10.8		
Backward inclination	40.9 ± 10.8		
**LBP duration**			
Less than 3-months (non-chronic)	40.1 ± 10.5	-4.56	0.000**
3-months and above (chronic)	45.1 ± 11.2		
**LBP severity**			
Mild	40.2 ± 10.5	3.27	0.039*
Moderate	42.7 ± 11.7		
Severe	42.2 ± 10.5		
**Sciatica**			
Yes	42.0 ± 11.8	0.86	0.389
No	41.0 ± 10.7		

*SD* standard deviation; ** *p* < 0.001; * *p* < 0.05

The Shapiro–Wilk test was performed prior to fitting linear regression model to test the normality of distribution of the dependent variable (OSD scores) and showed that *p* = 0.106, suggesting that the OSD scores were normally distributed. Based on the simple linear regression analysis (Table 4), the univariate model showed that age category 18 – 27 (β = 0.09, *p* = 0.038), being a female (β = 0.35, *p* < 0.001), heavy smoking (more than 11 cigarettes per day) (β = 0.12, *p* = 0.006), manual labour (β = 0.09, *p* = 0.032), chronic low back pain (β = 0.19, *p* < 0.001), abnormal illness behavior (β = 0.12, *p* = 0.006), and fear avoidance beliefs about work (β = 0.20, *p* < 0.001) and physical activity (β = 0.19, *p* < 0.001) were positively associated with disability, while only moderate smoking (β = -0.09, *p* = 0.042) was negatively associated with disability, which means moderate smoking was a protective factor. In the multivariable linear regression model (Table 5), the variables that significantly contributed to greater disability were the age category 48 – 57 years (β = 0.09, = 0.021) and fear avoidance beliefs about work (β = 0.34, *p* < 0.001). The female sex significantly contributed to less disability (β = -0.35, *p* < 0.001) Surprisingly smoking 1 to 10 cigarettes per day (β = -0.11, *p* = 0.004) and abnormal illness behavior (β = -0.14, *p* = 0.038) significantly contributed to less disability. The model accounted for 20% of the total variance in Oswestry disability scores.

**Table 4:** Correlation between continuous variables and OSD score in patients with LBP

Variable	r	*p*-value
Illness behaviour	0.17	0.006*
**Fear avoidance belief**		
Work subscale	0.20	0.000**
Physical activity subscale	0.19	0.000**

*SD* standard deviation, *r* Pearson’s correlation coefficient, *OSD* Oswestry disability score; ** *p* < 0.001; * *p* < 0.05

**Table 5:** Linear regression analysis showing factors associated with disability (OSD scores) in patients with LBP

	**Univariate analysis**			**Multivariate analysis**		
**Variable**	Beta (β)	95% CI	*p*-value	Beta (β)	95% CI	*p*-value
**Age** (*38 – 47*)						
18—27	0.09	0.29 – 10.25	**0.038**			
28—37	-0.04	-3.43 – 1.10	0.311			
48—57	0.08	-0.12 – 3.86	**0.065**	0.09	0.33—3.95	0.021*
58 +	-0.01	-2.20 – 2.11	0.745			
**Gender** (*Male*)						
Female	0.35	5.92 – 9.38	**0.000**	-0.35	-9.59—(-6.15)	0.000**
**Exercise** (*No exercise*)						
Frequent exercise	-0.08	-3.67 – 0.04	**0.055**			
**Cigarette smoking (***No smoking*)						
1 to 10 cigarettes	-0.09	-4.39 – (-0.08)	**0.042**	-0.11	-4.91—(-0.97)	0.004*
More than 11 cigarettes	0.12	0.94 – 5.56	**0.006**			
**Type of work** (*Semi-sedentary*)						
Sedentary	0.07	-0.46 – 4.97	**0.108**			
Manual work	0.09	0.10 – 2.09	**0.032**			
**Sitting posture** (*Backward inclination*)						
Straight back	0.08	-0.18 – 5.70	**0.065**			
Stooped	0.06	-0.57 – 4.06	**0.140**			
Forward inclined	-0.08	-3.81 – 0.01	**0.051**			
**LBP duration** (*less than 3-months*)						
3-months and above (chronic)	0.19	2.83 – 7.13	**0.000**			
**LBP severity** (*Mild*)						
Moderate severity	0.08	-0.05 – 4.06	**0.055**			
Severe LBP	0.04	-1.11 – 3.56	0.303			
**Illnes_Behaviour_Score**	0.12	0.03 – 0.19	**0.006**	-0.14	-0.26—(-0.01)	0.038*
**Fear avoidance beliefs**						
Work subscale	0.20	0.17 – 0.42	**0.000**	0.34	0.32—0.72	0.000**
Physical activity subscale	0.19	0.27 – 0.68	**0.000**			

*Italics* – variables excluded from the equation,

**Bold** – variables included in the multivariate analysis (significant at *p* < 2),

^*^variables significant at *p* < 0.05 in the multivariate model,

^**^variables significant at *p* < 0.001 in the multivariate model

## Discussion

The purpose of this study was to determine the factors associated with disability among adult low back pain individuals presenting at primary public hospitals in KwaZulu-Natal, South Africa. In this study, the prevalence of CLBP was 22.2%. The prevalence of LBP with severe symptoms was 18.8% (of which 58.7% had chronic low back pain), while moderate pain and sciatica were 26.5% and 19.1%, respectively. Based on the multivariable linear regression analysis: being a female and fear avoidance beliefs about work significantly contributed to greater disability, while moderate smoking and abnormal illness behavior significantly contributed to less disability. Based on the multiple comparison test (Bonferroni correction) heavy smokers (≥ 11 cigarettes/day) and manual work were significantly associated with severe disability.

The mean OSD score in this study was 41.2 ± 10.9 which was more than three times higher than what was reported by Yi et al. [35]. In this study we found that, the female sex was associated with disability. This was also in line with Yi et al. [35], who reported a significant association between sex and disability. However, most studies on CLBP disability do not report on the predictive effects of sex [19, 36–38]. Our study found no association between alcohol consumption and disability. Surprisingly, moderate smoking was significantly associated with disability. This contradicts with some recent studies, which found a positive correlation between smoking and low back pain disability [39–41]. Doualla et al. found conflicting results, reporting a significant association between alcohol consumption and disability, and no association between cigarette smoking and disability [19]. These differences could be attributed to the differences in social norms, health awareness, health literacy and accessibility of healthcare services in different countries [3, 8]. This can be reflected by the differences in the prevalence of smoking reported in previous studies [42–44]. The prevalence of smoking has been reported to be 27% in South Africa [44] and 11.2% in Cameroon [43]. Differences in the prevalence of smoking among provinces in South African have also been reported by Fagbamigbe et al. [45]. The Western Cape (37.9%) reported the highest lifetime prevalence of smoking, followed by the Northern Cape (33.2%) and Free State (30.7%) [45]. KwaZulu-Natal (20.2%) ranked the 6^th^ out of a total of 9 provinces in South Africa [45].

Psychological factors are known to influence disability in CLBP [19, 46–50]. We found that fear avoidance beliefs about work was significantly associated with disability. This concurs with several other international studies conducted in HICs [51–53]. A in Cameroon reported that, higher psychological wellbeing scores were significantly associated with less disability [19]. However, this disagreed with findings of the current study which reported that, abnormal illness perception contributed significantly to less disability. Doualla et al. did not investigate the influence of fear avoidance beliefs and illness behavior in their study [19]. Based on the findings of our study, qualitative occupational based studies should be conducted to investigate work related psychosocial factors that influence disability. In our study, based on the multiple linear regression analysis, we found that fear avoidance beliefs about physical activity does not influence disability. However, this association was significant on bivariate analysis. Fear avoidance beliefs about work, can potentially result in significant loss in production, decreasing the gross domestic product (GDP) [54]. Therefore, CLBP disability is burdensome at individual, community, and national level. Identifying risk factors early can help to reduce the incidence rate, which will eventually reduce the prevalence, economic burden and years lived with disability.

This study also found that, manual work was significantly associated with severe disability. This is potentially due to poor manual handling techniques caused by limited access to automated production systems, lack of strong policies such as the ‘no lifting policy’, lack of CLBP disability awareness, delayed detection of mild disease due to the general poor health seeking behavior, lack of qualified personnel who are trained to diagnose and treat chronic MSKD in KwaZulu-Natal. This concurs with what was observed by Gcelu et al., who reported that, in order to improve outcomes of MSKD in South Africa, there is need to allocate more resources for the training of personnel qualified to diagnose and treat these MSKD [13]. Gcelu et al. also reported that, “medical schools have to introduce students to the diagnosis and treatment of MSKD disorders early, general physicians and other specialists need to have greater exposure to these conditions and primary care workers should be trained to manage these MSKD disorders” [13]. The risky effects of manual work which is related to physical injury (be it microtrauma) has been well documented and is where the biomedical model of management stems [20]. However, due to the multifactorial nature of CLBP, this model (biomedical) alone failed to explain the mosaic of the pathophysiology of CLBP disability [55]. Thus, evidence has shown limited effectiveness of this traditional approach to management [20].

The management of CLBP should be based on a holistic multidisciplinary biopsychosocial approach. Based on the findings of our study, psychological factors (fear avoidance beliefs about work) do play a significant role in predicting disability in CLBP. Major-Helsloot et al. also reported role of psychosocial factors in influencing CLBP disability [14]. According to Major-Helsloot et al., the current management of LBP in South Africa is ineffective and not following the guidelines [14]. Another study reported that, the ineffective management of MSKD in South Africa could be attributed to limited medication options due to unaffordability and unavailability [13]. This could also be due to limited local data on risk factors for chronicity and the use of international guidelines without cross-cultural validation. A recently published user-friendly guideline in South Africa, based its recommendations on studies conducted in high-income-countries (HICs) [56], hence the need for local data. Thus, this study is important and calls for urgent attention for policy reconsideration in South Africa. Therefore, to improve outcomes of CLBP in South Africa, there is need for a focus shift, where more resources are allocated towards non-communicable diseases. Medical schools should introduce students to the diagnosis and treatment of CLBP early. Primary health care workers should be trained to identify warning signs of chronicity and to manage CLBP using conservative means. Resources should be allocated for the training of more chiropractors, rheumatologists, manual therapists, physiotherapists, exercise therapists, psychologists, psychotherapists, and social workers. Making available and accessible evidence-based information to healthcare providers and implementing measures to ensure adherence to guidelines.

The high prevalence of disabling back pain is a major concern to the public health system, hence it is particularly important to understand the predictive factors associated with CLBP disability. New policies and context-specific guidelines should be considered in the primary healthcare setting. These should include routine screening of predictors of CLBP disability, accessibility, and availability of qualified personnel especially in underserved communities. This satisfies the objective of the national development plan, vision 2030 [17]. Moore et al. in their study reported that the addition of a very brief cognitive behavioral therapy (CBT) to usual care from primary healthcare providers has shown to reduce pain and anxiety [57]However, according to Gatchel et al., CBT does not address all the factors that potentially contribute to disability in CLBP, such as the biological factors, hence a multidisciplinary approach is recommended [58]. Therefore, routine screening of risk factors for CLBP in primary care is highly recommended in South Africa to ensure early identification of warning signs for chronicity [21, 59]. This is because, delays in appropriate treatment can further contribute to disability as the window of opportunity to start treatment will have been missed. Thus, the use of a risk stratification tool (STarT Back) as part of all new patient screening in primary care should be adopted in South Africa in order to reduce the burden of CLBP disability.

### Strengths and limitations

To the best of our knowledge, this is the first study to be done in South Africa that seek to determine the factors associated with disability in CLBP, therefore, it fills that knowledge gap. This is an important study in this context as the burden of CLBP is continuing to increase, the factors that influence this are important to understand in order to inform policy on prevention and management. In line with the national development plan, this study serves as a wakeup call to policy makers and other involved stakeholders to draw attention to CLBP, the leading driver of disability. However, there are several limitations to this study such as the cross-sectional nature of the study methodology and the use of self-report questionnaires. Therefore, we cannot afford to exclude recall bias. Again, this study was conducted on participants presenting to the hospital for other ailments for healthcare, which means this might also have exaggerated our findings, making it difficult to generalize. Therefore, more cohort or community-based studies are needed to give a general picture of CLBP disability within the community.

## Conclusion

Evidence from this study has confirmed the influence of psychosocial factors at predicting disability in CLBP. Fear avoidance beliefs about work were significantly associated with greater disability. Heavy cigarette smoking and manual work were also identified as significant predictors of CLBP disability in this study. Considering the heterogeneity in population dynamics in South Africa, similar studies should be conducted in other districts/provinces to better generalize the findings, before examining potential cost-saving or cost-effective solutions. However, this does not take away the fact that, this study provides evidence-based knowledge that guides the development of context-specific efficacious guidelines to improve health outcomes and alleviate CLBP disability in KwaZulu-Natal, South Africa.

## Supplementary Information

**Additional file 1. **Age Stratified Demographic Characteristics.

## Data Availability

All data generated or analysed during this study will be included in the published article.
