# Supplementary material for: A cross-sectional hospital-based study of correlates of disability in patients with chronic low back pain in KwaZulu-Natal, South Africa
Source: BMC Musculoskelet Disord. 2022 May 11;23:438. doi: 10.1186/s12891-022-05397-4 (PMC9092723; doi:10.1186/s12891-022-05397-4)
Supplement: Supplementary file 1 — Additional file 1. Age Stratified Demographic Characteristics. [file 12891_2022_5397_MOESM1_ESM.docx]

| Characteristic | Categories | Overall (N = 544) | | Chronic LBP (n = 123) | |
| --- | --- | --- | --- | --- | --- |
|  |  | **(n)** | **(%)** | **(n)** | **(%)** |
| Gender |  |  |  |  |  |
| 18 – 27 | Males  Females | 15  4 | 2.7  1.0 | 4  2 | 3.3  1.6 |
| 28 – 37 | Males  Females | 44  67 | 7.9  12.1 | 4  17 | 3.3  13.8 |
| 38 – 47 | Males  Females | 74  102 | 13.4  18.4 | 16  19 | 13.1  15.5 |
| 48 – 57 | Males  Females | 68  95 | 12.3  17.2 | 12  22 | 9.6  17.9 |
| 58+ | Males  Females | 27  58 | 4.9  10.5 | 9  18 | 7.3  14.6 |
| Marital status |  |  |  |  |  |
| 18 – 27 | Single  Married  Separated  Widowed | 3  9  4  3 | 1.0  1.6  1.0  1.0 | 1  3  2  0 | 1.0  2.4  1.6  0.0 |
| 28 – 37 | Single  Married  Separated  Widowed | 13  58  27  14 | 2.3  10.5  4.9  2.5 | 5  9  4  3 | 4.1  7.3  3.3  2.4 |
| 38 – 47 | Single  Married  Separated  Widowed | 22  85  55  14 | 4.0  15.3  9.9  2.5 | 4  18  10  3 | 3.3  14.6  8.1  2.4 |
| 48 – 57 | Single  Married  Separated  Widowed | 28  74  46  15 | 5.1  13.4  8.3  2.7 | 6  21  4  3 | 4.9  17.1  3.3  2.4 |
| 58+ | Single  Married  Separated  Widowed | 14  20  25  6 | 2.5  3.6  4.5  1.1 | 4  14  8  1 | 3.3  11.4  6.5  1.0 |
| Body Mass Index (BMI) |  |  |  |  |  |
| 18 – 27 | Underweight  Normal  Overweight  Obese | 2  3  7  7 | 1.0  1.0  1.3  1.3 | 0  0  2  4 | 0.0  0.0  1.6  3.3 |
| 28 – 37 | Underweight  Normal  Overweight  Obese | 13  29  47  22 | 2.3  5.2  8.5  4.0 | 2  0  11  8 | 1.6  0.0  8.9  6.5 |
| 38 – 47 | Underweight  Normal  Overweight  Obese | 12  50  57  57 | 2.2  9.0  10.3  10.3 | 0  3  18  14 | 0.0  2.4  14.6  11.4 |
| 48 – 57 | Underweight  Normal  Overweight  Obese | 14  53  61  35 | 2.5  9.6  11.0  6.3 | 1  7  18  8 | 1.0  5.7  14.6  6.5 |
| 58+ | Underweight  Normal  Overweight  Obese | 10  31  30  14 | 1.8  5.6  5.4  2.5 | 4  2  13  8 | 3.3  1.6  10.6  6.5 |
| Level of Education |  |  |  |  |  |
| 18 – 27 | No formal  Primary  Secondary  Tertiary | 4  6  6  3 | 1.0  1.1  1.1  1.0 | 1  2  2  1 | 1.0  1.6  1.6  1.0 |
| 28 – 37 | No formal  Primary  Secondary  Tertiary | 13  44  29  25 | 2.3  7.9  5.2  4.5 | 3  6  2  10 | 2.4  4.8  1.6  8.1 |
| 38 – 47 | No formal  Primary  Secondary  Tertiary | 37  56  48  35 | 6.7  10.1  8.7  6.3 | 11  6  10  8 | 8.9  4.8  8.1  6.5 |
| 48 – 57 | No formal  Primary  Secondary  Tertiary | 27  63  43  30 | 4.9  11.4  7.8  5.4 | 7  10  6  11 | 5.7  8.1  4.8  8.9 |
| 58+ | No formal  Primary  Secondary  Tertiary | 25  25  18  17 | 4.5  4.5  3.2  3.1 | 10  6  3  8 | 8.1  4.8  2.4  6.5 |
| Income category |  |  |  |  |  |
| 18 – 27 | Poor  Low-emerging-middle  Emerging-middle  Realised-middle  Upper-middle | 4  9  1  4  1 | 1.0  1.6  0.1  1.0  0.1 | 2  2  1  1  0 | 1.6  1.6  1.0  1.0  0.0 |
| 28 – 37 | Poor  Low-emerging-middle  Emerging-middle  Realised-middle  Upper-middle | 23  61  19  5  3 | 4.2  11.0  3.4  1.0  1.0 | 6  10  3  1  1 | 4.8  8.1  2.4  1.0  1.0 |
| 38 – 47 | Poor  Low-emerging-middle  Emerging-middle  Realised-middle  Upper-middle | 26  96  28  12  14 | 4.7  17.3  5.1  2.2  2.5 | 8  16  2  4  5 | 6.5  13.0  1.6  3.3  4.1 |
| 48 – 57 | Poor  Low-emerging-middle  Emerging-middle  Realised-middle  Upper-middle | 34  71  29  19  10 | 6.1  12.8  5.2  3.4  1.8 | 13  9  5  3  4 | 10.6  7.3  4.1  2.4  3.3 |
| 58+ | Poor  Low-emerging-middle  Emerging-middle  Realised-middle  Upper-middle | 17  36  19  9  4 | 3.1  6.5  3.4  1.6  1.0 | 7  12  7  1  0 | 5.7  9.8  5.7  1.0  0.0 |
| Exercise |  |  |  |  |  |
| 18 – 27 | No  Yes | 13  6 | 2.3  1.1 | 5  1 | 4.1  1.0 |
| 28 – 37 | No  Yes | 62  49 | 11.2  8.8 | 14  7 | 11.4  5.7 |
| 38 – 47 | No  Yes | 97  79 | 17.5  14.3 | 24  11 | 19.5  8.9 |
| 48 – 57 | No  Yes | 101  62 | 18.2  11.2 | 29  5 | 23.6  4.1 |
| 58+ | No  Yes | 65  20 | 11.7  3.6 | 21  6 | 17.1  4.9 |
| Smoking |  |  |  |  |  |
| 18 – 27 | No  Yes, 1 – 10  Yes, ≥ 11 | 8  6  5 | 1.4  1.1  1.0 | 0  2  4 | 0.0  1.6  3.3 |
| 28 – 37 | No  Yes, 1 – 10  Yes, ≥ 11 | 72  16  23 | 13.0  2.9  4.2 | 5  4  12 | 4.1  3.3  9.8 |
| 38 – 47 | No  Yes, 1 – 10  Yes, ≥ 11 | 96  46  34 | 17.3  8.3  6.1 | 8  8  19 | 6.5  6.5  15.5 |
| 48 – 57 | No  Yes, 1 – 10  Yes, ≥ 11 | 101  36  26 | 18.2  6.5  4.7 | 10  11  13 | 8.1  8.9  10.6 |
| 58+ | No  Yes, 1 – 10  Yes, ≥ 11 | 47  22  16 | 8.4  4.0  2.9 | 7  7  13 | 5.7  5.7  10.6 |
| Alcohol |  |  |  |  |  |
| 18 – 27 | No  Yes, occasionally  Yes, frequently | 7  5  7 | 1.3  1.0  1.3 | 2  1  3 | 1.6  1.0  2.4 |
| 28 – 37 | No  Yes, occasionally  Yes, frequently | 54  34  23 | 9.8  6.1  4.2 | 6  5  10 | 4.9  4.1  8.1 |
| 38 – 47 | No  Yes, occasionally  Yes, frequently | 85  43  48 | 15.3  7.8  8.7 | 8  7  20 | 6.5  5.7  16.3 |
| 48 – 57 | No  Yes, occasionally  Yes, frequently | 79  45  39 | 14.3  8.1  7.0 | 10  9  15 | 8.1  7.3  12.2 |
| 58+ | No  Yes, occasionally  Yes, frequently | 39  21  25 | 7.0  3.8  4.5 | 4  8  15 | 3.3  6.5  12.2 |
| Type of work |  |  |  |  |  |
| 18 – 27 | Semi-sedentary  Sedentary  Manual | 7  3  9 | 1.3  1.0  1.6 | 0  2  4 | 0.0  1.6  3.3 |
| 28 – 37 | Semi-sedentary  Sedentary  Manual | 66  24  31 | 11.9  4.3  5.6 | 3  9  9 | 2.4  7.3  7.3 |
| 38 – 47 | Semi-sedentary  Sedentary  Manual | 106  20  50 | 19.1  3.6  9.0 | 3  10  22 | 2.4  8.1  17.9 |
| 48 – 57 | Semi-sedentary  Sedentary  Manual | 91  28  44 | 16.4  5.1  7.9 | 3  11  20 | 2.4  8.9  16.3 |
| 58+ | Semi-sedentary  Sedentary  Manual | 41  15  29 | 7.4  2.7  5.2 | 1  11  15 | 1.0  8.9  12.2 |
| Sitting posture |  |  |  |  |  |
| 18 – 27 | Straight back  Stopped  Forward inclined  Backward inclined | 5  5  6  3 | 1.0  1.0  1.1  1.0 | 0  3  3  0 | 0.0  2.4  2.4  0.0 |
| 28 – 37 | Straight back  Stopped  Forward inclined  Backward inclined | 13  15  37  46 | 2.3  2.7  6.7  8.3 | 4  7  9  1 | 3.3  5.7  9.3  1.0 |
| 38 – 47 | Straight back  Stopped  Forward inclined  Backward inclined | 20  29  68  59 | 3.6  5.2  12.3  10.6 | 7  8  16  4 | 5.7  6.5  13.0  3.3 |
| 48 – 57 | Straight back  Stopped  Forward inclined  Backward inclined | 15  34  50  64 | 2.7  6.1  9.0  11.6 | 5  12  14  3 | 4.1  9.8  11.4  2.4 |
| 58+ | Straight back  Stopped  Forward inclined  Backward inclined | 5  23  28  29 | 1.0  4.2  5.1  5.2 | 3  10  9  5 | 2.4  8.1  7.3  4.1 |
| LBP severity |  |  |  |  |  |
| 18 – 27 | Mild  Moderate  Severe | 10  6  3 | 1.8  1.1  1.0 | 0  3  3 | 0.0  2.4  2.4 |
| 28 – 37 | Mild  Moderate  Severe | 61  27  23 | 11.0  4.9  4.2 | 3  7  11 | 2.4  5.7  8.9 |
| 38 – 47 | Mild  Moderate  Severe | 96  52  28 | 17.3  9.4  5.1 | 1  19  15 | 1.0  15.5  12.2 |
| 48 – 57 | Mild  Moderate  Severe | 95  40  28 | 17.1  7.2  5.1 | 2  15  17 | 1.6  12.2  13.8 |
| 58+ | Mild  Moderate  Severe | 41  22  22 | 7.4  4.0  4.0 | 0  12  15 | 0.0  9.8  12.2 |
| Sciatica |  |  |  |  |  |
| 18 – 27 | Yes  No | 4  15 | 1.0  2.7 | 2  4 | 1.6  3.3 |
| 28 – 37 | Yes  No | 26  85 | 4.7  15.3 | 3  18 | 2.4  14.6 |
| 38 – 47 | Yes  No | 30  146 | 5.4  26.4 | 5  30 | 4.1  24.4 |
| 48 – 57 | Yes  No | 31  132 | 5.6  23.8 | 9  25 | 7.3  20.3 |
| 58+ | Yes  No | 15  70 | 2.7  12.6 | 3  24 | 2.4  19.5 |
